# INDACO project: a pilot study on incidence of comorbidities in COPD patients referred to pneumology units

**DOI:** 10.1186/2049-6958-8-28

**Published:** 2013-04-03

**Authors:** Giorgio Fumagalli, Fabrizio Fabiani, Silvia Forte, Massimiliano Napolitano, Paolo Marinelli, Paolo Palange, Antonella Pentassuglia, Stefano Carlone, Claudio Maria Sanguinetti

**Affiliations:** 1 Pulmonary Department of San Filippo Neri General Hospital, Rome, Italy; 2 Pulmonary Department of San Giovanni-Addolorata General Hospital, Rome, Italy; 3 Pulmonary Department of San Giovanni Battista Hospital, Rome, Italy; 4 Pulmonary Department of Institute of Internal Medicine, “La Sapienza” University, Rome, Italy; 5 Quisisana Clinical Center, Rome, Italy; 6 UOC Pneumologia, A.C.O. San Filippo Neri, Via Martinotti, 20, Rome, 00135, Italy

**Keywords:** BMI, Charlson comorbidity index, Comorbidities, COPD, COPD exacerbation, FEV_1_, Inhaled drug therapy, Smoking

## Abstract

**Background:**

Chronic Obstructive Pulmonary Disease (COPD) is often associated with comorbidities, especially cardiovascular, that have a heavy burden in terms of hospitalization and mortality. Since no conclusive data exist on the prevalence and type of comorbidities in COPD patients in Italy, we planned the INDACO observational pilot study to evaluate the impact of comorbidities in patients referred to the outpatient wards of four major hospitals in Rome.

**Methods:**

For each patient we recorded anthropometric and anamnestic data, smoking habits, respiratory function, GOLD (Global initiative for chronic Obstructive Lung Disease) severity stage, Body Mass Index (BMI), number of acute COPD exacerbations in previous years, presence and type of comorbidities, and the Charlson Comorbidity Index (CCI).

**Results:**

Here we report and discuss the results of the first 169 patients (124 males, mean age 74±8 years). The prevalence of patients with comorbidities was 94.1% (25.2% of cases presented only one comorbidity, 28.3% two, 46.5% three or more). There was a high prevalence of arterial hypertension (52.1%), metabolic syndrome (20.7%), cancers (13.6%) and diabetes (11.2%) in the whole study group, and of anxiety-depression syndrome in females (13%). Exacerbation frequency was positively correlated with dyspnea score and negatively with BMI. Use of combination of bronchodilators and inhaled corticosteroids was more frequent in younger patients with more severe airways obstruction and lower CCI.

**Conclusions:**

These preliminary results show a high prevalence of comorbidities in COPD patients attending four great hospitals in Rome, but they need to be confirmed by further investigations in a larger patients cohort.

## Background

Chronic obstructive pulmonary disease (COPD) is a major cause of morbidity and mortality and constitutes a heavy social and economic burden both in industrialized and emergent countries [1]. COPD is primarily characterized by airflow limitation not fully reversible due to airways inflammation and remodelling and to parenchymal destruction, but it is commonly associated with many extrapulmonary manifestations likely due to systemic inflammation [2,3].

Comorbidities are common in COPD patients, but the exact prevalence has not been established yet [4], because it is difficult to distinguish between extrapulmonary effects of COPD and true comorbidities. As it is known, COPD is an inflammatory disease, mainly induced by smoking and environmental pollution, that can lead to the pulmonary damage. Recently, according to other investigators, this pulmonary inflammation has also been thought to have a systemic effect, so playing a role in other pathologic conditions, such as muscle wasting, osteoporosis and cardiovascular alterations, frequently observed in patients with severe COPD [5].

The origin of systemic inflammation is not completely clarified yet. Some authors have postulated that the systemic inflammation could be related to a “spill-over” of lung inflammatory products into the circulation, leading to damage of different body structures [2]. On the other hand, it has been hypothesized that the pivotal factor is a systemic inflammatory state, mainly caused by smoking and other noxious agents, that determines the compromise of multiple organs, included the lung [6,7]. However, neither hypothesis has been definitively confirmed yet.

Furthermore, the relationships between comorbidities and COPD are rather complex due to smoking, aging, undesiderable drug effects and diagnostic inaccuracy, that can influence both the lung disease and the comorbidity itself [4]. Despite this uncertainty, many COPD patients present chronic medical conditions that have the feature of comorbidities, because the onset seems to be before the respiratory disease manifests or apparently unrelated to it: cardiovascular diseases, systemic hypertension and diabetes are the most frequent age-related diseases.

Largely, patients with COPD are elderly and present a number of other chronic medical conditions, like coronary artery disease, diabetes mellitus, arterial hypertension and osteoporosis [4,8]. Comorbidities, especially cardiovascular diseases, are a major risk factor for hospitalization and death in COPD patients [9,10], with increase in healthcare costs as demonstrated by several authors [9,11-14] in large cohorts of COPD patients.

The prognostic role of comorbidities has been pointed out by Antonelli-Incalzi et al. [15] in patients discharged from hospital after an acute exacerbation of COPD, in which the 5-year mortality was predicted by right ventricular hypertrophy, signs of myocardial infarction or ischemia and chronic renal failure, other than a FEV_1_ value below 600 ml and age. In another study the presence of ventricular arrhythmias and atrial fibrillation was an independent predictor of 1-year mortality [16].

The prevalence of comorbidities in COPD varies considerably across different studies [4,9,17-23]. Concerning Italy, a population-based retrospective study on data drawn from the Health Search database of Italian General Practitioners (GPs) found an increased risk for cardiovascular events [24] in COPD patients. Another longitudinal Italian study in acute exacerbations of COPD patients has evaluated the higher incidence of comorbidities and their burden in terms of hospitalization and death [25].

The aim of this study (INDACO: ***INDA****gine sulle****CO****morbidità nella BPCO*) was to assess the prevalence of comorbidities in COPD patients referred to pulmonary units of four major general hospitals in Rome, and to search correlations between prevalence and type of comorbidities with patients’ clinical and respiratory function characteristics. Here we report on the data of a first sample of patients.

## Methods

### Study design

This is an observational study, carried out in a defined time period of 12 months, without a control group.

Patients of both sexes, aged more than 40 years and with a diagnosis of COPD, obtained with clinical history, respiratory functional tests and radiologic exams, referred to the pulmonary wards of four major general hospitals in Rome, Italy, were enrolled in this study. They were a part of daily users of respiratory outpatient units of San FilippoNeri General Hospital, San Giovanni-Addolorata General Hospital, Umberto I Clinical Center of La Sapienza University and San Giovanni Battista Rehabilitation Hospital, referred either for a first examination in order to make a diagnosis of COPD or for the follow up of an already diagnosed COPD. All consecutive patients with COPD examined on the first day of each week (or another previously established day) during the study period were enrolled for this study. Patients without respiratory functional test were excluded in data collection. No other exclusion criteria was applied.

### Data collection

After clinical and functional examination, the respiratory physicians filled in a specifically-designed computerized questionnaire with the following information for each patient: personal and anthropometric data; smoking habit; clinical history of COPD and number of acute exacerbations in the previous year; results of spirometry, arterial blood gas analysis, and 6-minute walking test (6MWT), performed during clinical examination or within 3 months; use of inhalation therapy and drugs used at home: 1) “naïve” (no inhaler therapy), 2) bronchodilator use only**:** long-acting beta-adrenergic (LABA), with or without long acting anti-muscarinic (LAMA), 3) LABA and Inhaled Corticosteroids (ICS),4) LABA plus LAMA plus ICS. The “Charlson Comorbidity Index (CCI) [26] was calculated for each patient according to comorbidities explicitly declared by the patient and confirmed by continuative use of therapies. Also other comorbidities were collected with the same method of direct refer and compatible drugs use.

### Statistical analysis

Data are reported as mean value ± SD (standard deviation from the mean).

The statistical analysis took into account the distribution of variables of respiratory function, anthropometric characteristics, acute exacerbations and comorbidities in relation to hospital distribution, smoking habit, and the reported inhalation therapy. Then, we looked for possible correlations between the number of acute exacerbations in the previous year and the level of bronchial obstruction and gas exchange impairment, CCI score, and inhalation therapy.

Finally, the distribution of comorbidities was assessed in the whole study group, and in relation to respiratory function and CCI.

The statistical significance was investigated with the analysis of variance for normally distributed groups and values of p<0.05 were considered statistically significant.

For some correlations not reaching statistical significance the observed trend was taken into account.

The Ethics Committee of involved hospitals was informed about this observational study, which required a patients’ informed consent, as usual form of routinely clinical activities.

## Results

**Figure 1:**
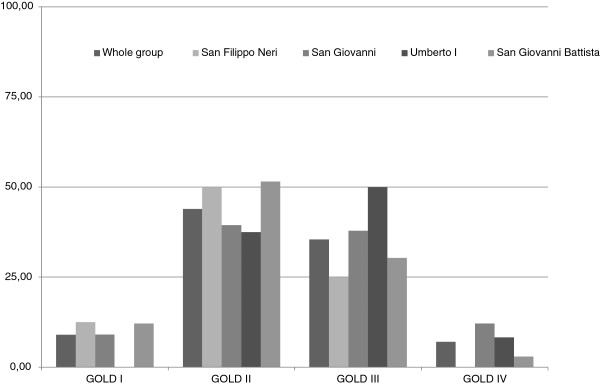
**Prevalence (%) of GOLD stages in 169 patients with COPD.** Local distribution of patients among Pneumology Centers measured by severity of functional respiratory impairment as FEV_1_ % grouping, according to 2010 GOLD guidelines.

From 1^st^ September 2009 to 30^th^ August 2010 we enrolled 169 patients, 124 males and 45 females, mean age 74 ± 8 years, all affected by COPD. Anthropometric and respiratory function characteristics are summarized in Table 1 for the whole sample and by each hospital pulmonary unit. The prevalence for each stage of the 2010’ GOLD classification is shown in Figure 1.

Mean BMI was 27.3±4.8, ranging from 26.6 to 28.5 and mean percentage of current smokers was 32.9%, ranging from 25% to 36.4% among the patients recorded in the four hospitals without significant differences. There were instead significant differences among the four hospital groups concerning the mean value of airways obstruction, as expressed by the FEV_1_/FVC ratio, number of acute exacerbations of COPD in the previous year, and score of the CCI (Table 1). In particular, the incidence of neoplastic comorbidities was significantly higher in two hospitals (San Giovanni-Addolorata General Hospital and Umberto I Clinical Center) than in the other two hospitals, thus increasing the CCI.

**Table 1:** Sample distribution as whole and by pneumology centers

	**Whole group (n = 169)**	**San Filippo Neri**	**San Giovanni-Addolorata**	**Policlinico Umberto I**	**San Giovanni Battista**	***p***
Age (mean ± SD)	74(± 8)	73(± 6)	74(± 10)	77(± 7)	72(± 6)	ns
Male (%)	72.8	74.3	74.0	50.0	84.8	ns
BMI (mean ± SD)	27.3(± 4.8)	28.2(± 4.9)	26.6(± 5.2)	26.8(± 4.1)	28.5(± 4.0)	ns
Current smokers (%)	32.9	31.4	34.7	25	36.4	ns
PaO_2_ mmHg (mean ± SD)	69.3(± 11.7)	71.6(± 13.6)	69.6(± 12.1)	66.8(± 8.9)	67.3(± 10.5)	ns
FEV_1_ % pred. (mean ± SD)	56.3(± 20.1)	65.7(± 21.4)	53.2(± 19.8)	50.4(± 15.4)	57.7(± 19.8)	0.01
FEV_1_/FVC % (mean ± SD)	54.6(± 14.5)	58.2(± 12.1)	55.9(± 12.7)	52.1(± 21.2)	49.8(± 14.7)	ns
COPD exacerbations (mean ± SD)	1.83(± 1.27)	1.02(± 1.3)	2.32(± 1.1)	1.37(± 1.1)	1.87(± 1.2)	< 0.001
Charlson Index (mean ± SD)	4.88(± 2.07)	3.62(± 1.8)	5.72(± 2.2)	5.33(± 1.7)	4.0(± 1.2)	< 0.001
Cancer %	13.6	5.7	22.1	12.5	3.0	ns
Lung cancer %	5.9	2.9	10.4	3.0	0	ns

**Figure 2:**
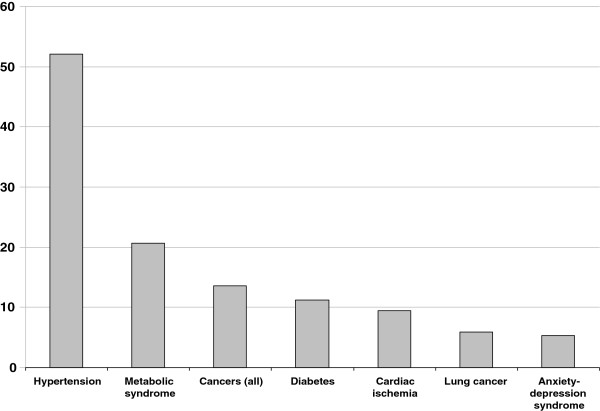
**Prevalence (%) of different comorbidities in 169 patients affected with COPD.** Global prevalence of observed comorbidities.

The number of patients presenting comorbidities was 159 (94.1%); 40 patients (25.2%) had only one comorbidity; 45 patients (28.3%) had two comorbidities, and 74 patients (46.5%) had 3 or more comorbidities. The mean prevalence of the different comorbidities in the whole sample of COPD patients is shown in Figure 2. In this preliminary group of investigation, a striking prevalence of systemic hypertension, as high as 52.1%, and - to a lesser extent - of metabolic syndrome (20,7%) and diabetes mellitus (11.2%) appears evident.

After stratification by the 2010’ GOLD stage, the prevalence of comorbidities showed a higher trend in the more severe stages, even if statistical significance was not reached (p = 0.08): 1.8 (SD ± 1.1) comorbidities for GOLD stage I, 2.2 (SD ± 1.3) comorbidities for GOLD stage II, 2.3 (SD ± 1.1) for GOLD stage III, and 2.5 (SD ± 1.1) for GOLD stage IV.

**Figure 3:**
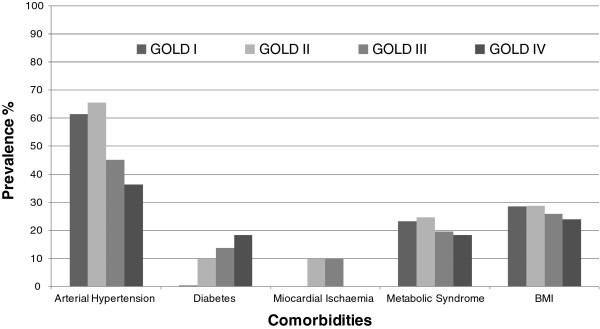
**Prevalence of comorbidities in COPD patients by GOLD stage and BMI value.** Global prevalence of comorbidities and Body Mass Index loss by severity of functional respiratory impairment as FEV_1_ % grouping, according to 2010 GOLD guidelines.

No significant correlation was observed between each type of comorbidity and GOLD stage, while BMI showed a significant inverse correlation with the worsening of COPD (Figure 3).

Current smoking was correlated with airways obstruction only as a trend when based on FEV_1_ value (52.45 ± 22.2 in smokers and 58.2 ± 18.7 in non smokers, p = 0.09), with statistical significance when FEV_1_/FVC ratio (51.1 ± 14.7 vs. 56.4 ± 14.1, p = 0.03) was considered. The mean age of current smokers was also significantly lower than that of non smokers (69.6 ± 7.5 years vs 76.1 ± 7.9, p < 0.0001). There was no correlation between smoking habit and prevalence of acute exacerbations of COPD or score of CCI.

No significant correlation was found between gender and BMI, age, airways obstruction, gas exchange, number of exacerbations or CCI score. In females, however, we found a significantly higher degree of dyspnea evaluated with modified Medical Research Council Dyspnea scale (mMRC=2.53 ± 0.9 in females vs 2.12 ± 0.8 in males, p = 0.001) and a higher incidence of anxiety-depression syndrome compared to males (respectively 13% vs 2.4%; CI 95% 0.03-0.69; p = 0.01), but no relation was found between this syndrome and airway obstruction or gas exchange.

**Figure 4:**
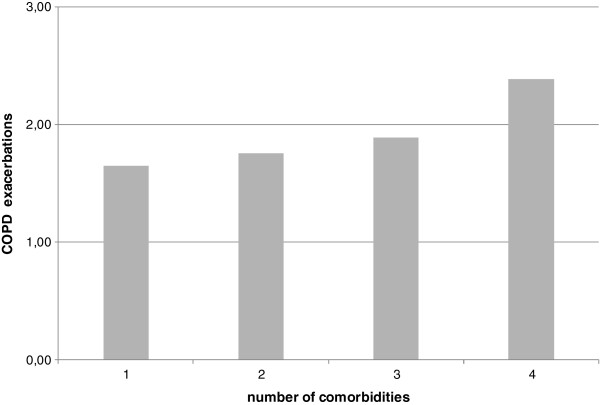
**Mean of COPD exacerbations by prevalence of comorbidities.** Link between comorbidities and acute exacerbations of COPD.

We observed a positive trend between the degree of airways obstruction and prevalence of acute exacerbations, level of hypercapnia, and CCI score. A progressive increase in number of exacerbations correlated with the increase in number of comorbidities, without significance at ANOVA (Figure 4). Moreover, the prevalence of acute exacerbations was positively correlated with dyspnea and negatively with BMI values (Table 2).

**Figure 5:**
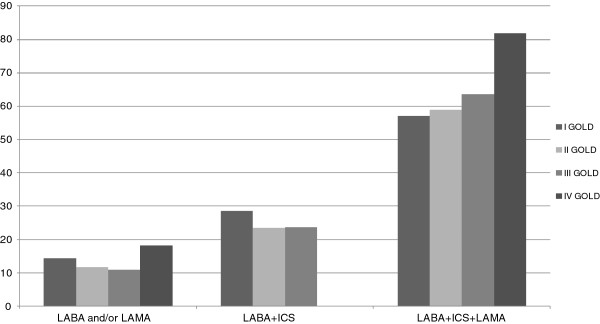
Complexity of inhalation therapy by respiratory function*. Distribution of inhalation therapy by respiratory dysfunction group. *GOLD staging 2010 was used to simplify the analysis of therapy prescription and because the data collection was performed before GOLD classification 2011.

According to inhaler therapy, patients were divided into 4 groups on the basis of whether they used only LABA and/or LAMA, or fixed combinations of LABA and ICS, or LABA plus ICS plus LAMA. No correlation was found between the number of comorbidities and the complexity of inhaler therapy (1, 2 or 3 inhaled drugs) even if a light trend was observed for 2010’ GOLD stage IV, the most severe stage of the disease (Figure 5). The use of three active drugs (LABA plus ICS plus LAMA) showed a significant positive correlation with severity of airways obstruction, and a negative correlation with age and CCI score. No relation was found between complexity of therapy and prevalence of acute exacerbations. Therapy with bronchodilators alone was significantly correlated with a lower degree of dyspnea (Table 3).

## Discussion

The real frequency and type of comorbidities in patients affected with COPD is not well established and a certain variability of results emerges from the different studies, although cardiovascular diseases seem most frequently associated with the chronic respiratory illness, probably because they share some risk factors.

In the present study we found a very high prevalence of comorbidities in COPD patients, more than 90%, greater than the prevalence reported in other clinical studies which ranged from 50% to 65% [21,23] and in administrative health services databases where the diagnosis of COPD was gleaned from the type of drugs used and duration of therapy [27]. The high prevalence of comorbidities in our cohort may be related to the high incidence of comorbidities, as casual correlation in our region or, more likely, to the evidence that about half of our patients were at stage GOLD III or IV, where, as well documented, there is a higher correlation with comorbidities.

**Table 2:** Sort of data by class of COPD exacerbation *

**Class of COPD exacerbation**	**Age (yrs)**	**FEV**_**1**_**%**	**FEV**_**1**_**/FVC %**	**PaO**_**2 **_**mmHg**	**PaCO**_**2 **_**mmHg**	**BMI Kg/m**^**2**^	**CCI score**	**MRC score**
0	73.8	63.7	58.5	73.2	37.8	28.1	3.9	1.7
1	73.7	56.8	56.7	70.3	41.4	29.1	4.9	2.4
2	74.9	54.9	52.5	69.8	44.4	26.9	5.1	2.1
3	72.9	51.4	52.9	66.4	44.5	25.9	5.2	2.5
*p*	ns	ns	ns	ns	ns	0.02	0.08	0.002

*(class 0 = 0 exacerbations; class 1 = 1 exacerbation/year; class 2 = 2 exacerbations/year; class 3 = 2 or more exacerbations/year).

In our patients cardiovascular diseases were the main pathological entities associated with COPD. In fact, arterial hypertension was found in more than 50% of patients and cardiac ischemia in about 10%. In a retrospective study [24] based on GPs’ records, the prevalence of heart diseases in COPD patients was found to range from 7.9% for heart failure to 13.6% for cardiac arrhythmias and to 23.1% for other forms of heart disease as a whole.

Compared to the latter study [24] the prevalence of arterial hypertension in our investigation (52.1%) was higher, and similar to that found in prospective investigations on patients hospitalized for acute exacerbation of COPD [23,25]. Data recorded in the general Italian population [28] reported a mean prevalence of hypertension of 33% in males and 31% in females, with the amount of borderline values ranging from 14% to 19%. We cannot distinguish between unequivocal hypertension and borderline forms, however our diagnosis of hypertension was based on the report of patients and on the drugs they assumed. Thus it is presumable that they were in the majority of cases truly affected by arterial hypertension. On the other hand, it is known that the odds ratio for hypertension is increased in COPD patients, particularly in those at higher stages of severity according to GOLD classification [10].

In our study, the prevalence of arterial hypertension, myocardial ischemia and metabolic syndrome was higher in GOLD class I and II, while only for diabetes we observed an incremental trend of prevalence with worsening of COPD (i.e. GOLD class III and IV). This finding may be explained by a wider presence of more compromised subjects in the higher GOLD stages, as reflected by a lower BMI. Moreover, in these patients it is more likely to observe diabetes (probably related to steroid therapy as a concomitant cause), while hypertension and metabolic syndrome, as diseases mainly associated with a status of wellbeing, decrease (Figure 3).

How COPD increases the risk of cardiovascular diseases and the poor outcome of these comorbidities is still not yet fully clarified. One possibility is that COPD patients have alterations of the neurohumoral regulatory system with increased sympathetic activation [29], that can be worsened by the use of beta-adrenergic bronchodilators [30]. Another hypothesis refers to the persistent low-grade systemic inflammation observed in COPD [5], that has a role in the formation and rupture of arterial plaque and in the onset of arteriosclerosis, with increased risk of ischemic heart disease, coronary alterations and stroke [31]. Furthermore, the increased arterial stiffness may predispose to systemic arterial hypertension and other cardiovascular diseases [32]. As a matter of fact, it has been demonstrated that the severity of respiratory impairment can predict the occurrence of cardiovascular disease [33].

**Table 3:** Sort of patients’ characteristics according to inhalation therapy

	**FEV**_**1**_**/FVC %**	**Age (yrs)**	**CCI score**	**MRC score**	**COPD exacerbations/year (mean)**
LABA and/or LAMA	52.3	74.8	4.8	1.9	1.61
LABA+ICS	59.9	76.8	5.5	2.5	1.78
LABA+ICS+LAMA	51.6	72.7	4.5	2.2	1.91
*p*	0.01	0.05	0.006	0.01	0.08

We also observed a high prevalence of metabolic syndrome in this preliminary sample of COPD patients, slightly lower than that predicted in the general population aged 60 years and over [34,35], probably due also to a prevalent number of males in our study. The prevalence of this pathologic condition was, however, higher than in other studies carried out in broad COPD populations, where it resulted very low [24], likely due to problems linked to the correct definition of metabolic syndrome and COPD based on the retrospective review of GPs’ databases. The metabolic syndrome is a complex of risk factors such as obesity, dyslipidemia, arterial hypertension and insulin resistance that predispose to diabetes and cardiovascular disease. Obesity is a major determinant both of metabolic syndrome and of systemic inflammation in the general population [36] and physical inactivity is a risk factor for it [37]. A recent investigation of the relationship between COPD and metabolic syndrome revealed that the prevalence of metabolic disorder is very elevated in COPD patients (about 50%), especially in the first two stages of the GOLD classification (probably because in stages III and IV there is a more conspicuous weight loss), associated with systemic inflammation and with the degree of physical inactivity [38].

The prevalence of diabetes was above 11% in our patients, which is higher than the mean percentage of 5% estimated in recent years in the Italian population but similar to that predicted for the over-60 years age-group [39]. The prevalence of diabetes that we observed may be mostly explained by the age of our subjects; however it has been observed that COPD patients have about double the relative risk of developing type II diabetes compared to normal subjects, due to the increase in circulating cytokines, in particular TNF-α, that interferes with glucose metabolism and insulin sensitivity [40,41].

Lung cancer is 3–4 times more frequent in patients with COPD, likely due to the increased inflammation and oxidative stress demonstrated in these patients [2,42]: in our investigation the prevalence of lung cancers was higher than that found in other studies [24,25] but in accordance with that in patients with moderate to severe airways obstruction [43].

Anxiety and depression are frequent in COPD patients, mainly because of the physical impairment that especially affects the most compromised patients, generally unable to maintain normal physical activities and social relationships. A condition of anxiety-depression was observed in more than 5% of our patients, a percentage lower than that reported in stable patients with severe COPD [44], probably because the great majority of our patients had moderate-severe COPD. Moreover, the anxiety-depression syndrome seems to have a different incidence in males and in females. Our COPD patients were mainly males; in fact, the prevalence of anxiety-depression syndrome increased to 13%, when only females were included in analysis.

The prevalence of acute exacerbation was found to correlate with airways obstruction, degree of dyspnea, lower BMI (with dyspnea and BMI the correlation was statistically significant) and with the frequency of comorbidities, while no correlation was found with the type and dose of inhalation therapy.

As expected, and in agreement with the guidelines for COPD, the use of three inhalation drugs was greatest in the most advanced GOLD stage, where the airways obstruction is very severe and all the available drugs are prescribed to improve patients’ health status.

Instead, we also found a negative correlation between complexity of inhalation therapy and age and CCI score: it may be that in the younger patients with a lower presence of comorbidities, the need to obtain the best results in terms of bronchodilation and general well-being exceeds the caution about using a treatment that has potentially dangerous side-effects.

## Conclusions

This study confirms the well known relationship between some comorbidities and COPD, but clarifies the prevalence of comorbidities in COPD in regional public health and real life setting. These preliminary results need however to be confirmed by further investigation in a larger cohort of patients.

Moreover, this study brings some light to the “dark” side of COPD therapy, that is not yet appropriatelyprescribed according to the respiratory functional and clinical status, that is worse obstruction or frequency of acute exacerbations.

Within this perspective, the study will be extended to other respiratory units with the aim of improving the understanding of COPD, related comorbidities and therapies.

## Competing interests

The authors declare that they have no competing interests.
